# The Effect of Group Music Therapy with Physical Activities to Prevent Frailty in Older People Living in the Community

**DOI:** 10.3390/ijerph18168791

**Published:** 2021-08-20

**Authors:** Feng-Ching Sun, Hui-Chi Li, Hsiu-Hung Wang

**Affiliations:** 1 Kaohsiung Municipal United Hospital, No. 976, Zhonghua 1st Rd., Gushan Dist., Kaohsiung City 804114, Taiwan; sfcmail333@yahoo.com; 2 College of Nursing, Kaohsiung Medical University, 100 Shih-Chuan 1st Rd., Sanmin District, Kaohsiung 80708, Taiwan; 3 College of Nursing, Asia University, 500 Lioufeng Rd., Wufeng, Taichung 41354, Taiwan; huichili@asia.edu.tw

**Keywords:** elderly, music therapy, frailty, physical fitness, cognition, depression

## Abstract

Background: The frail elderly are prone to falls and fractures, which can result in dependency, disability, admission to institutions, and even death. They are at increased risk of frailty due to decreased physical activity, cognitive decline, and depression. Some evidence suggests that music therapy with physical activities may be particularly beneficial. Objective: This study aimed to investigate the intervention effect of music therapy with physical activities (MTPA) on frail elderly in the community. Methods: A quasi-experimental design was adopted. We selected 10 community care centers in southern Taiwan, in which elderly people over the age of 65 were assigned to a MTPA group and a comparison group after obtaining their informed consent. The MTPA group performed group music activities once a week for 120 min for 12 weeks, while the comparison group only continued with their daily activities. Instruments in this study included the Kihon Checklist, Senior Fitness Test (with Body Mass Index (BMI) and seven physical fitness items), Mini-Mental Status Examination (MMSE), and Geriatric Depression Scale Short Form (GDS-SF). Results: A total of 132 community elders agreed to participate in this study, and 122 completed both the pretest and posttest, with 62 in the music therapy group and 60 in the comparison group. The results of ANCOVA showed that after intervention, except for BMI, the Kihon frailty assessment, seven fitness scores individually and in total, MMSE, and depression showed significant improvements in the music therapy group relative to the comparison group (all *p* < 0.05). Conclusion: MTPA can improve the frailty index, cognitive function, depression, and physical fitness index in the community elderly. The results of this study can be used as a reference for the design of activities for the community elderly, to provide them with appropriate activities, improve their physical functions, and improve or delay their disability.

## 1. Introduction

Population aging is a global issue, especially since the post-World War II baby boomers have reached old age. It is estimated that the 21st century will see the largest aging population in the history of mankind and that from 2025 to 2050, the elderly population will account for over 34% of the total population [1]. According to statistics, 15.57% of the total population in Taiwan is over the age of 65 [2], which is expected to reach 20% by 2026 [3]. Approximately 13.6% of the elderly over the age of 60 have increased frailty each year [2], with an annual incidence of 43.4 in 1000 people [4]. Declining fitness predisposes the frail elderly to adverse conditions, such as falls, fractures, dependency, disability, admission to institutions, and even death [5,6], as well as associated increases in healthcare costs [7], which have an impact on their families and society as a whole. Therefore, it is an important issue in the study of the elderly to explore ways to prevent the occurrence of elderly frailty and thus delay the progression of frailty.

Frailty is a clinical condition characterized by a reduction in the ability to maintain the reserve capacity of multiple physiological systems to a level beyond that of his or her chronological age, in which the body is unable to maintain homeostasis when external stressors arise, resulting in subsequent disability and other adverse outcomes. Fried, et al. [8] first described the concept of “frailty” as presenting the following clinical features: unintentional weight loss, self-reported exhaustion, weakness, slow walking speed, and low physical activity. Those with ≥3 of the above 5 symptoms are classified as frail, those with 1–2 symptoms are classified as pre-frail, and those without any symptoms are classified as robust [9]. Ensrud, et al. [10] simplified the above assessment to three symptoms (study of osteoporotic fractures, SOF): unintentional weight loss, inability to stand up from a chair five times without arm assistance, and reduced energy level. Those with ≥2 symptoms are classified as frail, those with one symptom are classified as pre-frail, and those without any symptoms are classified as robust.

Decline in physical activity, cognitive decline, or depression all tend to cause frailty. A systematic literature review by Chu, Chang, Ho and Lin [9] revealed that elders with depression are predisposed to frailty, while Horibe, et al. [11] suggest that slow walking speed, depressive tendency, and cognitive decline in community elders were associated with progression to pre-frailty or frailty. Frailty is significantly related to decline in upper-and lower-limb muscle strength and loss of balance, and loss of balance also increases the risk of frailty [12].

Physical activity intervention and music benefit physical function and mental health. A narrative review by Haider, Grabovac and Dorner [13] points out that most studies support the effectiveness of physical activity interventions on the reduction in frailty, and increase in physical performance and muscle strength. Shimizu, et al. [14] suggest that movement music therapy can ameliorate physical functions in older people under the age of 75. Jun, et al. [15] reveal that music-driven activities can improve shoulder joint and elbow flexion in stroke patients. Systematic literature reviews by Li, et al. [16] and Li, et al. [17] also indicate that medium- to long-term music therapies can ameliorate cognitive function and depression.

Physical activity, cognitive decline, and depression are closely related to frailty, each of which can be improved with the use of music therapy with physical activity (MTPA). However, few studies have examined the effectiveness of MTPA in preventing frailty among community elders. This study aims to investigate whether MTPA can improve physical fitness, cognitive function, and depression symptoms, thus exerting a positive impact on the frailty index in the community elderly.

## 2. Materials and Methods

### 2.1. Design

This study adopted a cluster quasi-experimental design to classify the participants into a MTPA group and a comparison group. A cluster quasi-experimental design—considering a single care center as a unit in grouping—rather than individual classification of participants was used in the grouping. This was performed in order to reduce the possibility of error with the presence of a participant in both the MTPA group and the comparison group in the same database. The MTPA group consisted of care centers that signed up for this study earlier, whereas the centers that joined later were assigned as in the comparison group. This assignment was used to reduce the possibility of error with the presence of a participant in both the MTPA group and comparison group in the same database. Data were collected before the intervention and after the 12-week session. The comparison group was offered this assignment intervention as compensation after the study was completed, and the intervention was found beneficial to the elderly.

### 2.2. Sample and Settings

The study site included 10 community care centers in southern Taiwan. Sampling was performed after providing the participants with an explanation of the study’s aims and methods. The inclusion criteria were: (1) aged 65 or above; (2) the ability to communicate in Mandarin or Taiwanese; (3) whether they attended 80% or more of the activities. The exclusion criteria were: (1) suffering from severe or acute cardiovascular, musculoskeletal, or pulmonary diseases and thus not recommended to perform exercise; (2) diagnosis of dementia (MMSE score ≤ 24); (3) unable to hear music. In this study, the sample size was calculated based on G power 3.1.1 version with the effect size of 0.3, a desired significance level of 0.05, two-tailed and a power of 0.8. The estimated minimal sample size was 90 participants, split into two groups.

**Figure 1:**
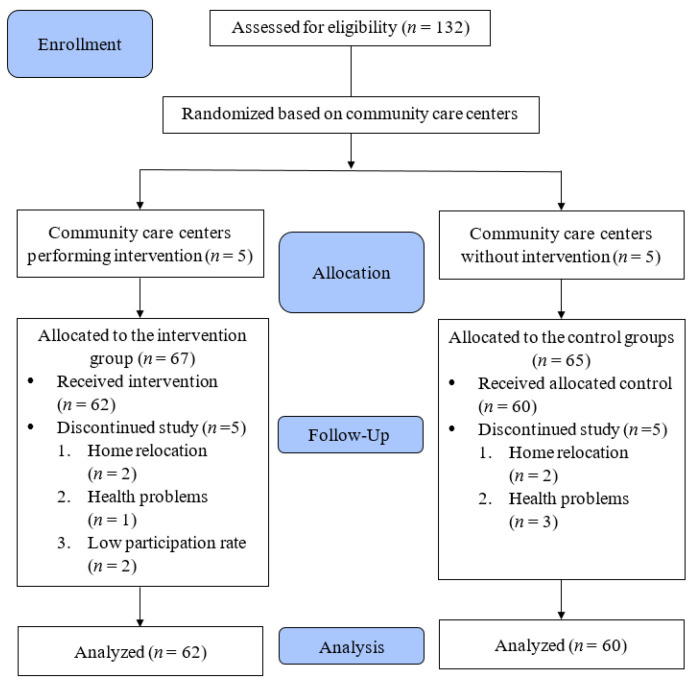
Flow chart of the participants through the study.

After the study was approved by the Institutional Review Board, a total of 132 community elders agreed to participate in the study. Informed consent was obtained from all of the subjects involved in the study. These elders were scattered across ten community centers, and the order in which the community care centers signed up for this study was used as a basis for grouping. The five community care centers that signed up first were the experimental group (*n* = 67) and the other five became the comparison group (*n* = 65). Ten community elders withdrew halfway through the study: four due to family relocation (*n* = 2 in the music therapy group; *n* = 2 in the comparison group), four due to health factors (*n* = 1 in the music therapy group; *n* = 3 in the comparison group), and two (in the music therapy group) due to a participation rate of less than 80%. In the end, 122 community elders completed the pretest and posttest, with 62 in the intervention group and 60 in the comparison group (Figure 1). The sample retention rate was 92%, with no significant difference between participants who withdrew and those who remained in the study with similar baseline data.

### 2.3. Intervention

The MTPA was an activity in which the participants listened to music and performed physical activity at the same time. The group music therapy group received 12 weeks of MTPA, with 1 120-min session per week. Twelve weeks of activities were divided into a relationship establishment period (Weeks 1 to 3), a functioning period (Weeks 4 to 10), and a task completion period (Weeks 11 to 12). A celebration was held in the last week of the program, and participants were encouraged to invite friends and relatives to join in the celebration to enhance their interaction with loved ones.

Two music therapists and a physiotherapist were involved in the design of the program for music arrangements and physical activities. Music genres included oldies, pop, nursery rhymes, and world-famous songs. The purpose of the design of the activities or selection of music was to enhance physical fitness, social interaction, life experience, and connection. A panel of experts from the academic and practical sectors (long-term care nurses, music therapists, sports therapists, and physiotherapists) were invited to conduct a content validity review after the design was finalized.

Each activity started with a warm-up, followed by a main body movement, and ended with a relaxation exercise, with a 10-min break between each part. Participants were advised to wear comfortable, breathable clothing and appropriate sports shoes to maintain comfort and safety. The instructor and assistant gave a brief greeting and performed simple health services and recorded blood pressure and temperature before each activity to confirm the condition of the participants.

To ensure effective observation and interpersonal interaction during the activity, the group size was kept under 18 participants, with an average of 13 people in each of the community care centers. The chosen music was familiar to the participants; in the case of new music, the subjects were guided to learn the rhythm of the track. The movements of each track were designed and reviewed by the physiotherapist to meet the functional activities of the participants. More specifically, simple instructions were used to ensure that the elderly participants could easily follow them and avoid frustration. The activities were designed to consider the functional limitations of the participants’ bodies, starting slowly and gradually increasing in speed, with simple movements for the big joints, and progressing to more detailed movements for daily use.

Data collection was conducted by four research assistants from nursing backgrounds who underwent two training sessions totaling 8 h to ensure the accuracy and consistency of the evaluation process. The comparison group followed their usual daily activities, such as simple hand-made crafts or chatting, with the exclusion of any type of group music activities.

### 2.4. Data Collection

Data were collected between August 2018 and March 2019, and the study tools included the Kihon Checklist, Senior Fitness Test, Mini-Mental Status Examination (MMSE), and Geriatric Depression Scale Short Form (GDS-SF).

#### 2.4.1. Kihon Checklist (KCL) 

The Kihon Checklist (KCL) is a Japanese care prevention checklist developed by the Ministry of Health Labour and Welfare [18], which is primarily used to screen the frail elderly [19]. Completion of the checklist has an approximate duration of 15 min and consists of 25 yes/no questions (yes = 0 point; no = 1 point) based on the following seven aspects: independent living, exercise, nutrition, oral cavity, social interaction, dementia, and depression. The lowest score is 0 and the highest is 25, with 0–3 indicating robust condition, 4–7 indicating pre-frail condition, and 8 or more indicating frail condition. A lower score is indicative of a good health condition [20]. Sentandreu-Mañó, et al. [21], as well as Yamada, et al. [22], have used this checklist to assess the frailty status of the community elderly. The reliability of the present study is represented by Cronbach’s α = 0.79.

#### 2.4.2. Senior Fitness Test

The test contains body mass index (BMI) and seven physical fitness items. The test has good reliability and validity and has been widely used in the assessment of the physical activity of the community elderly [23,24]. The intraclass correlation coefficient (ICC) of its test–retest reliability is between 0.93 and 0.98 [25].

The formula for calculating BMI is weight (kg) divided by the square of height (meters). The Health Promotion Administration recommends that the BMI for adults in Taiwan should be maintained between 18.5 (kg/m^2^) and 24 (kg/m^2^), as underweight (<18.5 kg/m^2^), overweight, or obesity (≥25.0 kg/m^2^) condition is detrimental to health. The seven physical fitness items [26] include: (1) lower-limb muscle endurance (chair-stand test: stand up repeatedly from a chair for 30 s; the participants start seated in the middle of a chair, with feet flat on the floor and arms crossed in front of the chest); (2) upper-limb muscle endurance (arm curl test: the participants sit on the edge of the chair on the side of the dominant hand, back straight, feet flat on the ground, dumbbell in the dominant hand, and upper arm clenched, to test the number of repetitions of elbow bending and straightening within 30 s); (3) cardiorespiratory endurance (2-min step test: stand in place and raise knees; count the number of steps taken in 2 min; mark with colored tape on the wall at half the distance between the participant’s patella and iliac femoral spine as the basis for knee elevation during stepping; and count the number of steps taken in 2 min); (4) lower-limb flexibility (chair sit and reach test: sit in chair and bend forward; bend one leg, straighten the other leg forward, heel on the ground, toes hooked; overlap hands up and down, reach toes to the extent possible, hold for 2 s, and take turns measuring 2 times on each leg; the scoring standard is to measure the distance between the fingertips and the toes and record with negative points; if the finger exceeds the toe, record with positive points, and choose the best score); (5) static body balance (one-leg balance with eyes open: stand on one leg with eyes open, with a full score of 120 s, hands on hips, the off-ground leg placed on the inner ankle of the supporting leg, to be performed alternatively on both legs); (6) upper-limb flexibility (back scratch test: place the dominant hand behind the shoulder on the same side, with the palm facing the back, the palm of the other hand extending from the lower back upwards, and the two hands should be as close as possible or overlapped. The scoring standard is to measure the distance between the 2 middle fingers, in which the inability to touch is recorded as a negative score and overlap as a positive score); and (7) functional mobility (timed ‘up & go’ test: sit on and subsequently stand from a chair surrounding objects to assess functional mobility and dynamic balance; the participants sit firmly in a chair, circles around the 2.44 m obstacle cone in front of them, and return to the chair; fewer seconds indicate better functional mobility).

#### 2.4.3. Mini-Mental Status Examination (MMSE)

Completion of MMSE requires approximately 5–10 min and is primarily used to assess cognitive function and contains 11 questions developed by Folstein, et al. [27], including items of orientation, attention, memory, speech, oral comprehension and behavior, and constructive ability. The full score is 30 points, with higher scores indicating improved cognitive function. One point is awarded for a correct answer; 20–23 points indicate mild cognitive impairment, 10–19 points indicate moderate cognitive impairment, 1–9 points indicate severe cognitive impairment, and 0 points indicate extremely severe cognitive impairment. The Cronbach’s alpha is 0.8 (Li, Chen, & Hsu, 2019).

#### 2.4.4. Geriatric Depression Scale-Short Form (GDS-SF)

The GDS-SF is used to identify depression in elders. It can be used with healthy, medically ill, and mild-to-moderately cognitively impaired subjects. The GDS-SF implemented in this study was a Chinese version translated by Chan [28] containing 15 questions, with a total score of 0–15 points. A higher score indicates a higher level of depression. The participants answer “Yes” or “No” to the questions based on experiences in the previous week, with “Yes” being awarded 1 point and “No” being awarded 0 points. A score of 0–6 indicates a good adjustment status, 7–10 indicates moderate emotional distress, and 11+ indicates severe emotional distress. The Cronbach’s alpha for this form was 0.89, the criterion-related validity was 0.95, and the concurrent validity was 0.96 [28]. The consistency of the present study is represented by Cronbach’s α = 0.72.

### 2.5. Data Analysis

SPSS 21.0 statistical package software was used for data input and analysis after data collection, and the significance level was set at 0.05. Descriptive statistics were used to analyze the mean value, standard deviation, frequency distribution, and percentages of the demographic characteristics of the participants, such as sex, age, education level, religious belief, and marriage. The χ^2^ test was used to check the association between the 2 categories of variables, or the *t*-test was used to check for significant differences between the mean values of the 2 groups. Eventually, ANCOVA was used to test the effectiveness of experimental interventions, including the Kihon Checklist, physical fitness, MMSE, and GDS-SF. In addition, when the ANCOVA was inserted, the collinearity test between the independent variables was performed first. If the independent variables were significantly correlated, they were not simultaneously used in the model.

## 3. Results

The χ^2^ test was used to examine the baseline data of the two groups. The results showed that the MTPA group was significantly different from the comparison group only in terms of gender, whereas the other variables were insignificantly different from each other (Table 1). In the MTPA group, 11.3% were male, compared with 33.3% in the comparison group (*p* = 0.003 < 0.05). These baseline data, especially gender, were subsequently used in the ANCOVA as covariates for analysis.

Table 2 shows the results of the interventions using independent samples and paired samples *t*-tests. Specifically, the mean values of the inter- and intra-group outcome items were examined to see if there were significant differences between the MTPA group and the comparison group. In the Kihon Checklist, only the MTPA group had a significant difference in the mean score of frailty in the pretest and posttest (decreased from 8.08 in the pretest to 5.84 in the posttest, *p* < 0.001). There were seven items in the physical fitness test, including lower-limb muscle endurance, upper-limb muscle endurance, cardiorespiratory endurance, lower-limb flexibility, static body balance, upper-limb flexibility, and functional mobility. Except for functional mobility, in which fewer seconds indicated better results, a higher value in the other items indicated improved function. Subsequently, a 5-point scale was used to assign each item a score from 1 (“not good”) to 5 (“very good”), which were added to the total fitness score. A comparison of the 2 groups revealed a significant difference between the pretest and posttest results: the MTPA group significantly improved, from 18.37 before the intervention to 21.95 after the intervention (*p* < 0.001), whereas the comparison group decreased significantly, from 16.45 to 15.93 (*p* < 0.001). Indeed, the comparison between the 2 groups showed that there was no significant difference in the pre-intervention score; however, the post-intervention score was appreciably higher in the intervention group than in the comparison group (*p* < 0.001). Therefore, preliminary results suggested that the music therapy group significantly improved after the music therapy intervention.

There was no significant difference in the total BMI score between the pretest and posttest results in the MTPA group; however, the comparison group showed a significant increase in mean ± SD from 23.82 ± 5.10 to 23.90 ± 5.09 (t = 3.113, *p* = 0.003).

The mean of the MMSE total score revealed a significant difference (t = 2.331, *p* = 0.021) between the MTPA group (M = 27.63, SD = 2.25) and the comparison group (M = 27.72, SD = 2.07) after the intervention, and the score in the intervention group changed appreciably before and after the paired sample t-test (from mean 26.74 to 27.63) (t = 3.935, *p* < 0.001).

In the geriatric depression assessment, only the MTPA group showed a significant difference in the mean frailty score in the pretest and posttest results (decreased from 2.18 in the pretest to 1.55 in the posttest, *p* < 0.001). This suggests that structured MTPA activities were effective in improving the cognitive function in the community elders, and that the effects of the MTPA intervention on frailty and depression in the elderly needed further analysis using ANCOVA.

Table 3 shows the effects of experimental intervention using ANCOVA. The demographic characteristics of participants showed that the music therapy group was significantly different from the comparison group only in terms of gender. Thus, after using sex as covariates, the results of ANCOVA analysis show that 12-week music intervention significantly improved in the Kihon Checklist (*F* = 63.509, *p* < 0.001), MMSE (*F* = 21.029, *p* < 0.001), but shows no significant effect on the elders’ BMI (*F* = 0.000, *p* = 0.997). Table 3 also shows that the music therapy group performed significantly better than the comparison group in all 7 items of physical fitness. Indeed, the 12-week music therapy intervention had a significant positive effect on upper- and lower-limb muscle endurance, cardiorespiratory endurance, upper- and lower-limb flexibility, body balance, and functional mobility and depression (GDS-SF) (*F* = 23.184, *p* < 0.001) in the community elderly.

## 4. Discussion

In terms of physical fitness, this study found that the music therapy with physical activity (MTPA) group had significantly better posttest scores relative to the pretest scores, whereas the comparison group had significant worse posttest scores relative to the pretest scores. A possible reason for the significant deterioration of physical fitness in the comparison group is their inactivity program, which caused the degradation of physical function [29]. Moreover, this study found that the MTPA groups showed significant improvements in the total physical fitness score and the score of each item compared to the comparison group. Music listening relieves pain and increases joint mobility during exercise [30], and familiar music tracks increase participation and retention rate in the elderly [31]. In this way, MTPA improves physical fitness by alleviating symptoms of discomfort during activities and boosting mobility and participation. These findings are similar to those found in Jun, Roh and Kim’s [15] study among hospitalized stroke patients, i.e., there was an improvement in range of motion (ROM) (shoulder flexion and elbow joint flexion) in the music-movement therapy group and a deterioration in the comparison group, and there existed marked differences between the groups. In a study among the community elderly, Shimizu, Umemura, Hirai, Tamura, Sato and Kusaka [14] also revealed that compared to the comparison group, the motor music therapy group had significantly improved body balance (one-leg balance with eyes open) and functional mobility (timed ‘up & go’ test).

Regarding BMI, there was no significant change in the MTPA group, but an appreciable increase in the comparison group was found when the pretest and protest results were compared. Moreover, there was no significant difference in BMI between the MTPA group and the comparison group. This may be due to the fact that the subjects of this study were elderly with slow metabolism, thus 12 weeks of MTPA had little effect on the weight of the participants. The impact of MTPA therapy on BMI in the elderly has been rarely studied, and it has been suggested that the effect be investigated in future studies by increasing the frequency or extending the duration of music therapy with physical activity.

The frailty assessment of the community elderly in this study significantly improved after MTPA therapy, whereas no significant improvement was observed in the comparison group, when pretest and protest results were compared. Moreover, the MTPA group showed significant improvements in frailty compared to the comparison group. Few studies have investigated the effectiveness of MTPA on frailty; however, the present study assessed similar frailty indicators to the study of osteoporotic fractures (SOF) [10]: BMI and lower-limb muscle endurance (chair-stand test). The present study found no dramatic decrease in BMI in the MTPA therapy group but did find a significant improvement in lower-limb muscle endurance (chair-stand test) in the physical fitness domain, which means frail was prevented based on SOF indicator. Future studies should investigate the effectiveness of MTPA in delaying the frailty of the community elderly by targeting various aspects to reduce the adverse prognosis of dependency, disability, and admission to institutions [5,6].

The results of the present study suggest that cognitive function was significantly improved in the MTPA group, but not in the comparison group, when the results of the pretest and protest were compared. The MTPA group also did significantly better than the comparison group in terms of cognitive improvement. Previous research has also demonstrated that music therapy can improve cognitive function [17,32]. The possible reason is that our music with physical activity therapy itself is a pleasurable activity, and such an activity is designed to increase attention and stimulate memory [33]. Furthermore, the music used in musical activities in the present study was familiar to the elderly, which could also stimulate their memory of such music.

This study indicated that there was a significant improvement in depression in the MTPA group, but not in the comparison group, when pretest and protest results were compared. Furthermore, the music with physical activity therapy group showed significant improvement in depressive symptoms compared to the comparison group. Such results were similar to most previous studies. Jun, Roh and Kim’s [15] study showed that music-movement therapy improved depression in people with stroke. In the study by Dev, et al. [34], single-group pretest and posttest results revealed that music therapy relieves the symptoms of depression in institutionalized elderly people. Additionally, Li, Wang, Lu, Chen, Lin and Lee [16] and Chu, et al. [35] suggest that music therapy can improve depression in dementia. It is possible that music with physical actives therapy can alleviate depression, since the act of listening to music can alleviate the symptoms of depression [36], and the design of MTPA group in this study increased interaction among participants, thereby relieving depression [37,38]. Thus, participants in this study were able to listen to music and increase social interaction, thereby relieving their depressive symptoms.

### 4.1. Study Limitations

The intervention duration in this study was 12 weeks. Although there were significant effects on frailty, physical fitness, cognitive function, and depression, there were no significant changes in BMI. Thus, we suggest that future studies extend the intervention duration or increase the activity frequency to observe changes in BMI. The present study was a quasi-experimental design study with too few male participants, and the sex ratio between the experimental group and the comparison group varied; thus, the inference of experimental results could be affected by gender. Different types of MTPA can be designed in future studies to recruit more male participants, and a random control trial design can be adopted to increase the validity of the results.

### 4.2. Relevance to Clinical Practice

The results of this study may be helpful for selection of activities for the community elderly, more specifically, music therapy with physical activity, which can improve frailty, physical fitness, cognitive function, and depression among the elderly, exerting a potential indirect effect on delaying disability and dementia in the community. Moreover, the results can be used as a basis to provide suitable activities for the community elderly, improve or delay dementia, and activate physical functions in this population, and can also be used as a reference for activity design for the community elderly.

## 5. Conclusions

The findings in this study indicate that MTPA can improve frailty, physical fitness, cognitive function, and depression among the community elderly, thereby providing a good direction for activity design for the community elderly. Nevertheless, since there were no significant improvements in BMI, we suggest that future studies should increase the activity frequency to investigate the effect of music therapy with physical activity on body weight and plan diversified activities to recruit more elderly males.

## Figures and Tables

**Table 1:** Basic demographics.

Variable	Total	Intervention Group (*n* = 62)		Comparison Group (*n* = 60)		*p* Value
		*n*	(%)	*n*	(%)	
**Sex**						0.003
Male	27	7	(11.3)	20	(33.3)	
Female	95	55	(88.7)	40	(66.7)	
Age (years, mean ± SD)	73.90 ± 6.86	74.29 ± 7.53		73.50 ± 6.14		0.631
**Education level**						0.168
Illiteracy	33	15	(24.2)	8	(13.3)	
Primary school/literate	49	26	(41.9)	23	(38.3)	
Junior high school and above	50	21	(33.9)	29	(48.3)	
**Religious belief**						0.744
Yes	10	6	(9.7)	4	(6.7)	
No	112	56	(90.3)	56	(93.3)	
**Marital relationship**						0.144
Yes	49	29	(46.8)	20	(33.3)	
No	73	33	(53.2)	40	(66.7)	
Children						0.356
Yes	5	4	(6.5)	1	(1.7)	
No	117	58	(93.5)	59	(98.3)	
**Living with family and friends**						0.369
Yes	24	14	(22.6)	10	(16.7)	
No	98	48	(77.4)	50	(83.3)	
Health behavior						0.234
Yes	115	2	(3.2)	5	(8.2)	
No	7	60	(96.8)	56	(91.8)	
Number of illnesses (mean ± SD)	2.87 ± 2.31	3.23 ± 2.20		2.50 ± 2.52		0.092
**Physical illness**						0.722
Yes	13	6	(9.7)	7	(11.7)	
No	109	56	(90.3)	53	(88.3)	

Notes: 1. This table was based on χ^2^ test; 2. Age and number of illnesses were determined using independent samples *t*-test.

**Table 2:** Comparison of pre- and post-intervention effectiveness between the two groups.

Variable	Intervention Group (*n* = 62)	Comparison Group (*n* = 60)	*t*	*p* Value
	Mean ± SD	Mean ± SD		
**Kihon Checklist**				
Pre-intervention	8.08 ± 4.82	6.57 ± 3.67	1.845	0.068
Post-intervention	5.84 ± 4.15	6.65 ± 3.76	−1.130	0.261
Paired *t*-test (*p* value)	8.144 (<0.001)	−1.692 (0.096)		
**Total physical fitness score**				
Pre-intervention	18.37 ± 7.47	16.45 ± 4.30	1.748	0.084
Post-intervention	21.95 ± 7.26	15.93 ± 4.11	5.654	<0.001
Paired *t*-test (*p* value)	−7.961 (<0.001)	4.305 (<0.001)		
BMI				
Pre-intervention	23.58 ± 3.51	23.82 ± 5.10	−0.309	0.758
Post-intervention	23.67 ± 3.49	23.90 ± 5.09	−0.291	0.772
Paired *t*-test (*p* value)	0.904 (0.369)	3.113 (0.003)		
**MMSE total score**				
Pre-intervention	26.74 ± 2.45	26.88 ± 1.96	−0.353	0.725
Post-intervention	27.63 ± 2.25	26.72 ± 2.07	2.331	0.021
Paired *t*-test (*p* value)	3.935 (<0.001)	−1.932 (0.058)		
**Geriatric depression**				
Pre-intervention	2.18 ± 2.24	2.10 ± 2.30	0.188	0.851
Post-intervention	1.55 ± 1.52	2.10 ± 2.00	−1.719	0.088
Paired *t*-test (*p* value)	−4.608(<0.001)	0.000 (>0.999)		

**Table 3:** Effects of music therapy on frailty, MMSE, BMI, physical fitness, and depression in the community-dwelling elders.

	Group (Ref. = Comparison Group)						
	Pre-Mean		Post-Mean		F	*p* Value	Effect Size
Variable	Intervention	Comparison	Intervention	Comparison			
Kihon posttest score	8.08	6.57	5.20	7.31	63.509	<0.001	0.350
Posttest BMI (kg/m^2^)	23.58	23.82	23.78	23.78	0.000	0.997	<0.001
Posttest total score of physical fitness	18.37	16.45	21.21	16.70	89.260	<0.001	0.431
Physical fitness sub-items							
1 Posttest lower limb muscle endurance (times)	13.61	14.00	17.20	13.26	63.261	<0.001	0.349
2.Pretest upper limb muscle endurance test (times)	18.74	16.27	20.67	17.08	29.679	<0.001	0.201
3 Cardiorespiratory endurance (times)	66.48	78.57	83.40	71.59	18.263	<0.001	0.134
4 Posttest lower limb flexibility-right (cm)	−1.95	−2.47	0.84	−2.47	53.289	<0.001	0.311
5 Posttest lower limb flexibility-left (cm)	−1.61	−3.12	0.53	−2.92	41.138	<0.001	0.259
6 Posttest static body balance-right (s)	11.32	16.13	18.78	13.09	16.503	<0.001	0.123
7. Posttest static body balance-left (s)	10.36	12.41	16.32	11.53	8.148	0.005	0.065
8. Posttest upper limb flexibility (cm)	−8.41	−8.53	−6.43	−8.81	17.483	<0.001	0.129
9. Posttest functional mobility (s)	13.11	10.68	10.44	12.52	63.878	<0.001	0.351
MMSE posttest score	26.74	26.88	27.72	26.63	21.029	<0.001	0.151
Geriatric depression posttest score	2.18	1.55	1.52	2.13	23.484	<0.001	0.166

## Data Availability

The data presented in this study are available on request from the corresponding author.
